# Post-Operative Delirium in Elderly Patients: A Narrative Review

**DOI:** 10.3390/ijms262311314

**Published:** 2025-11-22

**Authors:** Alexander Smirnov, Michael Semionov, Valery Yasinski, Yair Binyamin, Alexander Zlotnik, Dmitry Frank

**Affiliations:** Department of Anesthesiology, Soroka University Medical Center, Faculty of Health Sciences, Ben-Gurion University of the Negev, 8410501 Beer-Sheva, Israel; smirnovalexander69@gmail.com (A.S.); semyonov.michael@gmail.com (M.S.); valeryyasinsky@gmail.com (V.Y.); yairben1@gmail.com (Y.B.); alexander.zlotnik.71@gmail.com (A.Z.)

**Keywords:** elderly patients, postoperative cognitive dysfunction, delirium, general anesthesia

## Abstract

Anesthesia in older patients is challenging and requires a range of skills in various techniques, both during surgery and in the post-operative period. Post-operative delirium is one of the most common cognitive dysfunctions after surgery, and elderly patients are at the highest risk. The pathophysiology of post-operative delirium remains incompletely understood. Several mechanisms (vascular, neurodegenerative, neuroimmune, neuroinflammation, drug-induced, stress-induced, and monoaminergic) have been considered to play a role. The type of anesthesia—general (gas, total intravenous), regional, or combined—was identified as a predictive factor. However, numerous prospective and retrospective studies have failed to determine which anesthetic technique is the best for preventing post-operative delirium. The type of surgery appears to be more critical than the type of anesthesia. However, the development of post-operative cognitive dysfunction could be linked to the depth of anesthesia. Dexmedetomidine displayed promising therapeutic potential for the efficient prevention or treatment of hyperactive post-operative delirium. The management of hypoactive post-operative delirium still requires further investigations, particularly in elderly patients.

## 1. Definition of the Problem

Currently, more than half a billion people worldwide are older than 65, and, according to the United Nations, this number is expected to reach one and a half billion by the end of 2050. The number of elderly patients who undergo surgery and anesthesia is also increasing. Along with the surgery, pre-existing comorbidities put these patients at a particular risk of developing post-operative cognitive decline (POCD) [1].

Postoperative delirium (POD) is an acute and severe complication, especially in elderly patients; it typically occurs one to three days after surgery, but it can occur shortly after the surgery or up to seven days later. A 2020 review by Jin et al. (BJA) reports that POD affects 20–50% of elderly patients after major surgery, compared to less than 15% in younger populations [2,3]. Post-operative care may require continued hospitalization in the intensive care unit (ICU) or geriatric department. In the ICU, the incidence of delirium in elderly patients may exceed 80%, especially after prolonged mechanical ventilation or sedation. POD is associated with a 30-day mortality of 7–10%, and, accordingly, the cost of hospitalization increases three to four times. POD patients are at an increased risk of death: an 11% increase in the first three months and up to a 17% increase within one year [4].

Depending on the symptoms, POD can manifest as hyperactive, hypoactive, or mixed. The hypoactive form is most common, occurring in 40–70% of delirium cases, and it has a 67.6% prevalence in the geriatric population. It is often underdiagnosed due to its subtle presentation (lethargy, withdrawal). It is associated with higher mortality and worse outcomes due to delayed recognition, manifested with inactivity, lethargy, drowsiness, and staring into space. Despite its quiet presentation, the prognostic consequences are poor, carrying the highest mortality rate among all subtypes of POD, about 40% [5,6].

Hyperactive POD is less common, occurs in 15–25% of cases, and is expressed in agitation, loss of consciousness, restlessness, confusion, disorientation, hallucination, memory loss, functional dependence, and impaired cognitive function. The mixed type of POD represents 15–35% of cases, manifesting as fluctuations between hypoactive and hyperactive features; it requires more complex treatments than hyperactive or hypoactive POD alone [7,8].

## 2. Risk Factors

There are numerous risk factors for POD. Aging, pre-existing cognitive impairment (such as dementia or mild cognitive impairment), sensory impairments such as hearing or vision loss, and pre-operative mental health status and cognitive function are especially critical. In addition, factors such as the type and duration of surgery, the depth and kind of anesthesia, perioperative blood loss, current medications (including polypharmacy), and perioperative pain management are key [9].

Furthermore, deep anesthesia, as indicated by a bispectral index (BIS) value less than 35, is associated with a significantly higher incidence of POD than BIS values greater than 50. Aging and comorbidities, such as dementia, cerebrovascular disease, post-transient ischemic attack (TIA), alcohol and drug dependency, and diabetes mellitus, could strongly promote the development of POD [10,11]. Age, cognitive decline, and use of opioids were predictive of POD that was associated with increased length of stay (median 12 vs. 7 days, *p* < 0.001) and six-month mortality (19% vs. 6% in non-delirious patients, *p* = 0.02) [11].

Indeed, the risk of developing POD is much higher in patients undergoing non-cardiac surgery when they reach ages over 65. In patients with baseline cognitive impairment, the prevalence of POD is also much higher than it is in patients who do not have any deterioration of cognitive function [12]. The type of surgical intervention significantly influences POD risk. The risk of POD increases in ascending order: abdominal surgery (10–20%), cardiothoracic surgery (up to 52%), and femoral fixation (up to 70%) [13]. Not surprisingly, emergency surgeries carry a significantly higher risk (20–45%) than elective ones (2–3%) [14]. A meta-analysis has shown that age, cognitive impairment, the type and urgency of surgery, and the depth of anesthesia are the strongest independent predictors of POD [15].

## 3. POD and Risk of Persistent Post-Operative Cognitive Decline in the Elderly

POCD refers to persistent deterioration in cognitive performance following surgical procedures, particularly in elderly patients. It typically affects aspects of memory, attention, processing speed, and executive function and can present days to weeks after surgery, sometimes persisting for months or even becoming permanent [16].

Unlike POD, which is acute and fluctuating, POCD is subtle, non-fluctuating, and often detectable only through formal neuropsychological testing. It is most commonly observed in patients over 60 and is associated with major surgeries, general anesthesia, inflammation, and perioperative cerebral hypoperfusion. POCD may lead to reduced functional independence, increased risk of dementia, and diminished quality of life [16,17].

POD is a well-established independent risk factor for the development of persistent POCD in older adults. Multiple cohort studies and prospective trials have demonstrated that elderly patients who experience POD are significantly more likely to suffer from long-term cognitive impairments compared to those without POD [17].

Marcantonio et al. [18] found that patients ≥ 70 years who developed POD after elective non-cardiac surgery had double the risk of cognitive decline at six months compared to delirium-free patients. Inouye et al. demonstrate a strong association between incident delirium and long-term cognitive decline, persisting up to 36 months postoperatively. Saczynski et al. [19] observed that elderly cardiac surgery patients with POD had significantly lower cognitive performance at one-month and one-year follow-up. The meta-analysis conducted by Goldberg et al. showed that delirium increases the odds of long-term cognitive impairment by up to three-fold, especially in patients with pre-existing cognitive vulnerability [20].

POD is not only a transient complication—it is a powerful predictor of long-term cognitive decline in the elderly. Prevention and early management of delirium are therefore critical not just for immediate outcomes, but also for protecting long-term mental health.

## 4. The Role of the Type of Anesthesia

There are two types of anesthesia: general (inhalational or total intravenous anesthesia (TIVA)) and regional (RA). In some cases, various types of anesthesia could be combined. Any anesthesia includes pre-, intra-, and post-operative care, which could contribute to the development and progression of POD. Each period of perioperative care is coupled with a higher risk of POD in elderly patients [21].

### 4.1. Pre-Operative Care

The primary aim of pre-operative care, especially in elderly individuals, is to normalize physiological and biochemical variables, such as blood pressure, heart rate, and glucose levels. The water and electrolyte balance, as well as the acid–base balance, requires particular care. In fact, any deviations from homeostasis could increase the risk of POD. For example, hypovolemia induced by pre-operative fasting may increase the incidence of POD. Another pre-operative risk factor is associated with premedication, i.e., with anxiolytic and stress relief drugs [22].

### 4.2. Intra-Operative Care

An essential question arises regarding anesthesia: can anesthesia change the incidence of POD? The role of anesthesia in the pathogenesis of postoperative delirium (POD) remains contentious. While some randomized controlled trials and meta-analyses suggest that regional anesthesia is associated with a reduced incidence of POD compared to general anesthesia, other large-scale studies, including RCTs such as the RAGA trial, fail to demonstrate a statistically significant difference between the two modalities. The REGAIN trial (NEJM, 2021), involving over 1600 elderly patients undergoing hip fracture surgery, found no significant difference in POD incidence between spinal and general anesthesia. However, a slight trend favored spinal anesthesia [23]. Similarly, a Cochrane meta-analysis conducted by Neufeld et al. concluded that the type of anesthesia is not a strong independent predictor of POD compared with other perioperative factors, such as surgical stress, patient frailty, or intraoperative hypotension [24].

Regional anesthesia (RA) has significant advantages compared to general anesthesia (GA). A recent meta-analysis shows that using peripheral nerve blocks or combining regional with GA reduces POD incidence (RR 0.42–0.46), likely due to markedly lower postoperative pain and opioid consumption [25,26].

In turn, general anesthesia is divided into inhalational anesthesia (IA) and total intravenous anesthesia (TIVA). There is no substantial evidence regarding the advantages of TIVA over IA in the development of POD. But some studies demonstrate the benefits of TIVA compared to GA. Propofol shows greater burst-suppression activity than Desflurane and Sevoflurane, but it does not correlate with the highest POD risk. A recent clinical trial showed that Desflurane maintenance has been associated with a 1.8-fold higher risk of POD development in geriatric patients compared to propofol. Thus, the inherent neurobiological and pharmacological characteristics of the specific IA, such as rapid offset and neuroinflammatory effects, must play an independent role in POD pathogenesis [27,28]. Some studies suggest that TIVA anesthesia may offer theoretical neuroprotective benefits compared with inhalational agents [29]. Daccache et al. (2025) found that TIVA might decrease agitation and POCD and increase recovery time [30].

Despite these controversies, today, we certainly know that the depth of anesthesia is a critical player in the progression of POCD. Now, the depth of anesthesia is difficult to measure correctly, and the depth of anesthesia plane is associated with an increased risk of POD. The incidence of POD in patients after anesthesia, with BIS-monitored depth of anesthesia at 16.7%, was 21.4% in the control group (*p* = 0.036) [31]. In the trial conducted by Sieber et al. [26], elderly patients receiving lighter sedation during spinal anesthesia had a significantly lower POD rate (19%) compared to those under deep sedation (40%). Similar results have been reported, indicating that BIS-guided anesthesia reduces anesthetic exposure and decreases the risk of POD by 35%. For every 1000 elderly patients undergoing major surgery, a BIS value between 40 and 60 prevented 83 patients from developing delirium [32]. A randomized controlled trial demonstrated that targeting BIS readings of 50 was associated with a decrease in cases of POD compared to BIS 35 (deep anesthesia) and cognitive impairment at one year [33,34].

## 5. Assessment Tools for Detecting POD

There are various scores for the assessment POD. The Diagnostic and Statistical Manual of Mental Disorders, Fifth Edition (DSM-5), published by the American Psychiatric Association, is the gold standard for diagnosing delirium [5]. According to the DSM-5, delirium is diagnosed when all the following criteria (A–E) are met (disturbance in attention and awareness, acute onset and fluctuating course, additional cognitive disturbance, not better explained by pre-existing neurocognitive disorder, evidence of a medical cause) with sensitivity of 84–100% and specificity of 77–100% [35,36].

Many tools can help detect POD. The leading assessment tool is the Confusion Assessment Method for the ICU (CAM-ICU), which demonstrated a sensitivity of ~100% and specificity of ~98% in the validation study conducted by Ely et al. [37], comparing CAM-ICU against DSM-IV criteria evaluated by psychiatrists.

Other commonly used diagnostic tools include the Intensive Care Delirium Screening Checklist (ICDSC), which is particularly effective for detecting hypoactive delirium, and the Richmond Agitation-Sedation Scale (RASS), which helps assess hyperactive forms of delirium. The ICDSC is an eight-item checklist that uses long-term observation, with a typical diagnostic threshold of >2. Chen et al. demonstrate that the ICDSC has a sensitivity of 0.83 and specificity of 0.87 compared with CAM-ICU, which achieved values of 0.84 and 0.95, respectively [36].

## 6. Pathogenesis of POD

**Figure 1:**
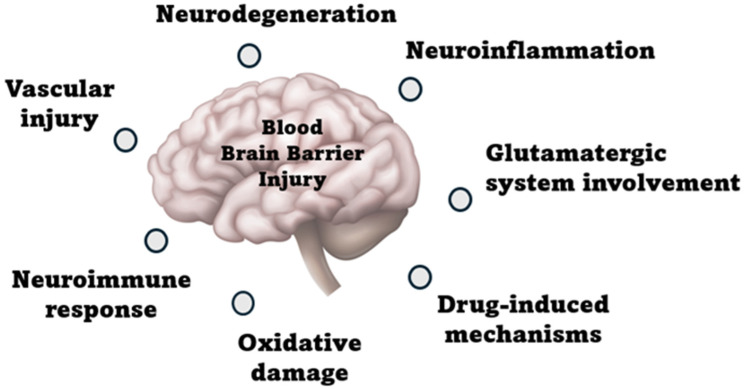
Pathophysiological mechanisms of post-operative delirium.

Delirium can develop at any age, but it is more frequent in the elderly because the brain’s functional reserve is much lower in older patients than in younger patients. Despite decades of research on POCD, its pathophysiology remains unknown. This review focuses on multiple POCD mechanisms, the effects of the type of anesthesia, and current treatments. However, various potential mechanisms for developing POCD exist, including neuroinflammation, neurodegeneration, neuroimmune response, oxidative stress, drug-induced effects, vascular involvement, and potential glutamatergic involvement, as shown in Figure 1 [38,39].

### 6.1. Neurodegeneration

**Figure 2:**
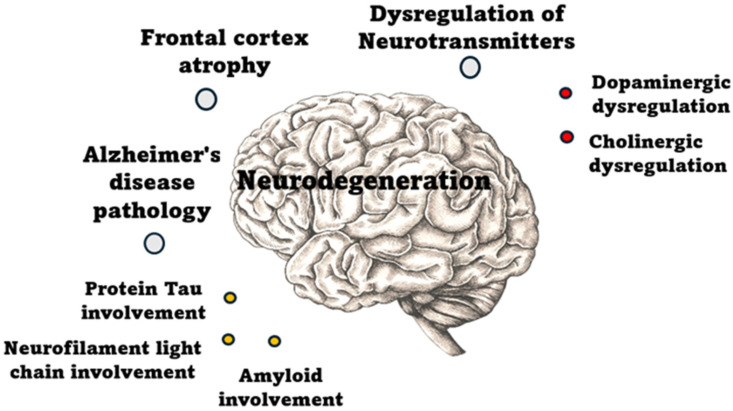
Pathophysiological mechanisms of neurodegeneration.

Neurodegeneration plays a key role in the pathogenesis of POD. Structural brain changes, such as frontal cortex atrophy and Alzheimer’s disease (AD) pathology, increase vulnerability to cognitive disturbances. Neurotransmitter imbalance, including dopaminergic and cholinergic dysfunction, further impairs neural signaling, contributing to the development of acute confusion and delirium. These changes may lower the threshold for POD, particularly in elderly patients with pre-existing neurodegenerative conditions, as shown in Figure 2.

#### 6.1.1. Frontal Cortex Atrophy

Frontal lobe dysfunction is associated with delirium. Notably, the bilateral ventrolateral frontal, orbitofrontal, and superior frontal cortices are involved in the pathogenesis of POD. Frontal lobe vulnerability contributes to the low cognitive reserve observed in older adults, making them more susceptible to disruptions from systemic stressors, such as surgery, anesthesia, or inflammation. Furthermore, frontal atrophy correlates with the severity and duration of delirium, as shown in ICU patients via volumetric MRI and atrophy network mapping studies [40]. Moreover, POD is followed by increased functional activity in the prefrontal and posterior cingulate cortex. The prolonged duration of delirium is correlated with damage to the superior frontal lobe in Intensive Care Unit (ICU) patients [41].

#### 6.1.2. Neurotransmitters’ Involvement in the Pathogenesis of Delirium

Basal forebrain neurons are the leading cholinergic producers of the central nervous system. Their atrophy could result in cognitive decline. However, the pathogenesis of psychotic behavior disturbances is associated with dysregulation of the cholinergic system. Of note, changes in the activity of acetylcholinesterase, butyrylcholinesterase, and choline acetyltransferase in CSF were seen in patients with POD, indicating central cholinergic degradation. Unfortunately, clinical studies have not demonstrated a positive effect of cholinergic inhibitors on POD [42]. The dopaminergic system is also involved in the development of POD. Stimulation of the nigrostriatal pathway may lead to the development of hallucinations [43]. Dopamine is an excitatory neurotransmitter involved in various physiological functions, including motor control, cognition, and the learning process. It is also a neuromodulator of Long-Term Synaptic Plasticity (LTP) and a leading neurotransmitter of the reward system. Regarding dopaminergic disruption of mesolimbic and mesocortical dopaminergic receptors and cognitive dysfunction, dopamine contributes to the appearance of psychosis [44,45]. In turn, it can appear as psychosis. Dopaminergic hyperactivity in the brain regions is responsible for the development of neuropsychiatric disorders that may cause symptoms of mania and psychosis. Thus, the involvement of cholinergic and dopaminergic systems may be critical in POD and warrant further investigation [45,46].

Neurotransmitter dysregulation—especially cholinergic deficiency and dopaminergic excess—has been strongly implicated in the pathophysiology of POD. Elevated anticholinergic activity and dopaminergic hyperactivity have been independently associated with delirium [47,48]. This is supported by clinical and biochemical studies, including those conducted by Plaschke et al. [49], Rossi et al. [50], and Meagher et al. [51].

#### 6.1.3. Alzheimer’s Disease Pathology: Involvement of Protein Tau, Neurofilament Light Chain, and Amyloid

Protein Tau, neurofilament light chain (NfL), and amyloid β are leading biomarkers for diagnosing Alzheimer’s disease in its early stages [52]. An increase in plasma Tau levels is associated with POD and its severity [53]. There is a decrease in CSF amyloid β1-42 in non-demented patients who developed delirium after hip fracture [54]. In turn, the cytoskeleton is composed of neurofilaments, which maintain the shape, size, and function of neurons. The elevation in NfL is indicated for axonal damage, which can be measured for the early recognition of delirium. Plasma NfL levels correlate with POD; patients with pre-existing neuronal damage are at higher risk of POD. Additionally, a relationship exists between the concentration of NfL and the severity of POD; pre-operative plasma with elevated NfL often accompanies severe delirium [55].

### 6.2. Neuroinflammation

Surgical tissue trauma triggers the release of damage-associated molecular patterns (DAMPs), which in turn initiate the activation of immune cells. In turn, the activation of neutrophils and monocytes accelerates the systemic inflammatory response, which targets the brain and other organs. HMGB1 is a prototypical DAMP that is released after surgical intervention and infection and causes neuroinflammatory responses in trauma. It can be recognized in plasma within 30 min after surgery. Tumor necrosis factor (TNF) and HMGB1 subsequently recruit bone-marrow-derived macrophages (BMDMs) into the inflammatory process. However, HMGB1 activation of the inflammatory process leads to the release of proinflammatory cytokines, such as interleukin (IL)-1β and IL-18. The complement system can be activated by DAMPs, as evidenced by increased C3 in synapses. Some studies have demonstrated altered C3 concentrations in CSF in delirium patients after hip fracture. Blood–brain barrier (BBB) injury is exacerbated by fibrinogen entry, which binds to complement receptor 3 (CR3) and activates macrophages/resident microglia, leading to cognitive deficits. Additionally, perivascular fibrinogen deposition was found in the hippocampus 24 h after surgery [56].

The central neural system (CNS) has immune cells, such as parenchymal (microglia) and nonparenchymal macrophages, which rapidly activate following brain injury. Proinflammatory cytokines are produced by microglia, such as TNF-α, IL-1ꞵ, IL-6, IL-8, and IL-12. Microglia can initiate A1 astrocytes by activating C1q, IL-1, and TNF, thereby exacerbating neuroinflammation [57,58].

The involvement of the blood–brain barrier initiates a systemic inflammatory response triggered by aseptic intervention. It disrupts endothelial cells, increasing BBB permeability to inflammatory cytokines and to peripheral blood macrophages. This starts a cascade of reactions that leads to neuroinflammation [59]. These inflammatory processes, in turn, contribute to the generation of reactive oxygen species (ROS), thereby amplifying oxidative stress.

### 6.3. Oxidative Stress

Reactive oxygen species (ROS) are free radicals produced by oxygen metabolism in mitochondria. The increase in ROS results from an imbalance between its overproduction and the body’s natural ability to eliminate it. In this manner, ROS contributes to delirium-like behavior following anesthesia and surgery. Brain tissue is highly vulnerable to oxidative stress due to its high metabolic activity and minimal antioxidative capacity, which contribute to Ca^2+^ cellular overload, lower levels of cytochrome c oxidase, and increased release of iron from injured brain cells, potentially driving the production of free radicals [60]. Moreover, ROS may interact with numerous biological molecules, including proteins, lipids, and deoxyribonucleic acid (DNA), thereby damaging cell membranes. This injury leads to protein aggregation, degradation, lipid peroxidation, and direct DNA damage, ultimately contributing to structural changes and mutations. ROS can open the mitochondrial permeability transition pore (MPTP), which causes mitochondrial swelling. Oxidative stress progression may lead to apoptosis and cell death [5,61,62].

### 6.4. Potential Role of the Glutamatergic System

Glutamate is a non-essential amino acid that acts as a neurotransmitter and accounts for nearly 60% of all neuro-mediatory excitatory activities contributing to cognition. When it is dysbalanced, it can promote neurologic and psychiatric disturbances. For example, cognitive impairment in schizophrenia can be the result of hypo-glutamatergic activity. In turn, hyperstimulation of glutamate receptors may cause excessive glutamate release, leading to neuronal death—a phenomenon known as “excitotoxicity” [63]. Sustainable brain activity in the prefrontal cortex is achieved through a coordinated and dynamically regulated balance between excitatory (mostly glutamatergic) and inhibitory (largely GABAergic) activities. When it is dysbalanced, it may cause neurodegenerative/psychiatric disorders and acute cognitive dysfunctions [64,65]. In turn, glutamate secretion may induce extreme neuronal excitability after surgery in mice, leading to neuronal damage and potentially delirium [64].

### 6.5. Vascular Mechanism

Age-related changes in the neurovascular unit impair microvascular circulation, leading to increased blood–brain barrier permeability, which represents the vascular component of POD. Age-related changes in cerebral autoregulation impair the brain’s ability to maintain consistent perfusion under hemodynamic stress (e.g., during anesthesia or hypotension), which contributes to the development of delirium. Intraoperative hypotension, hypoxia, and anemia reduce cerebral oxygen delivery, particularly in cortical watershed regions [66,67]. The vascular mechanism of POD highlights the role of cerebral hypoperfusion, microvascular ischemia, and blood–brain barrier dysfunction during delirium development. In particular, in elderly patients undergoing cardiac or vascular procedures, impaired neurovascular regulation and systemic inflammation increase vulnerability to acute brain injury.

Delirium after cardiothoracic surgeries requiring cardiopulmonary bypass (CPB), alongside stroke pathophysiology, reveals the vascular mechanism of POD, based on microemboli formation [68].

### 6.6. Drug-Induced Delirium

Numerous reviews suggest that various drugs have a deliriogenic potential, such as benzodiazepines, tricyclic antidepressants, opioids, and anticholinergics, which results in the alteration of γ-aminobutyric acid, noradrenaline, and decreased cholinergic transmission, leading to a hyperdopaminergic state [69].

Neurotransmitters play a crucial role in the pathogenesis of delirium. Acetylcholine is an excitatory neurotransmitter that contributes to various brain functions, including attention, memory formation, learning, and sleep [70]. Acetylcholine insufficiency can lead to delirium. Drugs with anticholinergic activity are often prescribed for elderly patients. Therefore, it may lead to cholinergic insufficiency, which in turn increases the risk of delirium. The pathogenesis is based on the blockade of M1 postsynaptic receptors, which are mainly located in the central nervous system (CNS) [71].

#### Beers Criteria: High-Risk Drugs and Their Influence on Delirium

The American Geriatrics Society (AGS) Beers Criteria identifies medications that are potentially inappropriate for use in the elderly due to their potential to cause adverse cognitive effects. Several of these drugs are strongly associated with delirium, especially in the postoperative setting [72]. Many drugs listed in the Beers Criteria are known to induce or worsen POD by interfering with cholinergic, GABAergic, or dopaminergic systems (Table 1). Regular medication review and avoidance of these agents—especially in frail or cognitively impaired elderly patients—are key components of delirium prevention strategies [73].

## 7. Management

**Figure 3:**
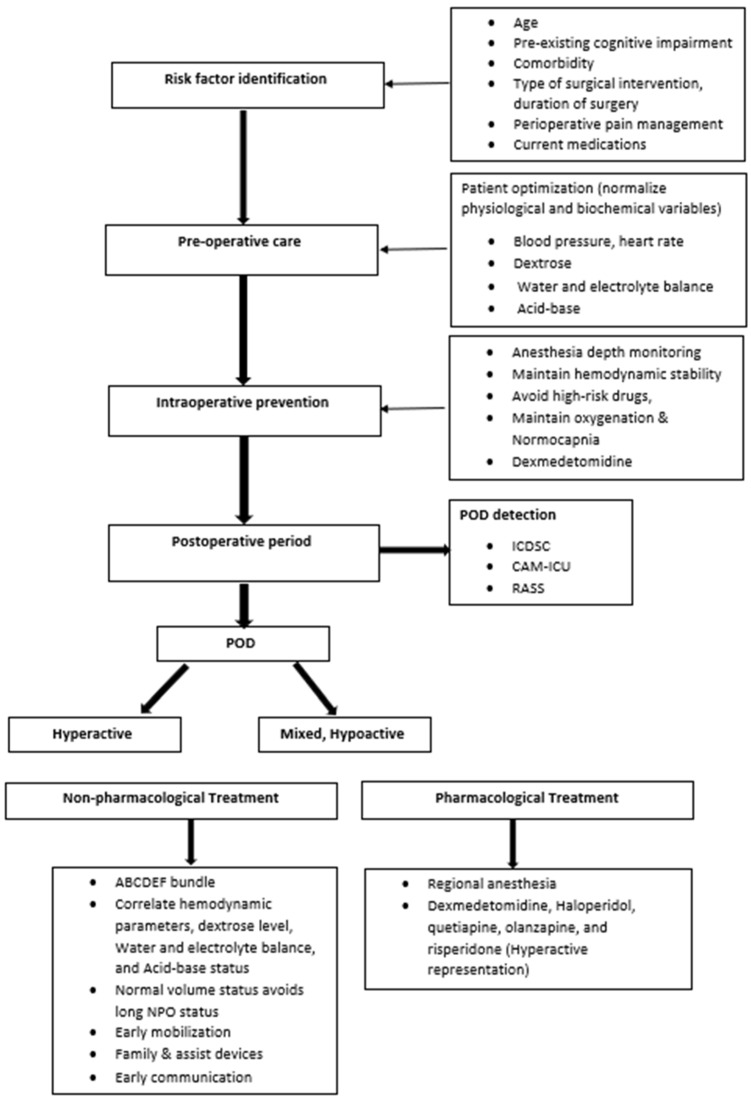
Perioperative management of POD.

The management of POD involves a multimodal strategy that focuses on identifying and treating underlying causes, minimizing contributing factors, and using pharmacological interventions only when necessary. Non-pharmacologic measures are the foundation of care. Orientation is maintained through the use of clocks, calendars, and regular staff reorientation. ABCDEF bundle application is used to optimize sensory input by ensuring patients have their glasses/hearing aids early after surgery. Sleep hygiene is promoted by minimizing nighttime disruptions, avoiding sedatives like benzodiazepines, and encouraging early mobilization and physical therapy. Additionally, family involvement is allowed when possible (Figure 3).

Maintaining stable hemodynamics and ventilation parameters intraoperatively is highly relevant to prevent hypotension and hypocapnia. Mean arterial pressure during surgery and partial pressure of carbon dioxide are physiologic variables related to POD because they significantly reduce cerebral blood flow. Duan et al. show that intraoperative hypotension (MAP < 65 mmHg) for extended durations was significantly associated with increased risk of POD [74]. In addition, Vasunilashorn et al. [75] demonstrated that variability in blood pressure and CO_2_ levels during surgery was associated with higher rates of POD, regardless of the type of anesthesia used. All this evidence suggests that maintaining stable hemodynamic and respiratory parameters is the best means of preventing POD, compared to lighter anesthesia. Preventative options included abstaining from giving psychotropic drugs or drugs that influence cognitive function, the existing strong recommendation of avoiding prolonged fluid fasting of more than 6 h [76]. Bogani et al. showed that ERAS elements, including liberal fluid intake up to 2 h before surgery, resulted in shorter hospital stays, fewer complications, and lower POD incidence in elderly cohorts [77].

After ruling out alternative causes or once the patient is clinically stable, pharmacological treatment may be considered for managing POD. Several medication options exist, including haloperidol, quetiapine, risperidone, and dexmedetomidine [78]. Haloperidol, a typical butyrophenone-type antipsychotic, acts by blocking dopamine receptors, which may lead to increased central acetylcholine levels [79]. It has anti-hallucinatory, anti-delusional, and anti-agitation effects [80]. Some small studies have shown that early prophylactic haloperidol administration may reduce the incidence of POD. However, it is not universally recommended as a first-line agent secondary to its side effect profile, which includes extrapyramidal symptoms such as acute dystonia and Parkinsonism, hyperthermia, and the rare but potentially fatal neuroleptic malignant syndrome [81,82].

Second-generation (atypical) antipsychotics such as quetiapine, olanzapine, and risperidone are associated with a lower risk of motor side effects due to their greater affinity for serotonin 5-HT2A receptors in addition to dopamine blockade [83]. In a randomized controlled trial, Grover et al. compared haloperidol and risperidone in delirium treatment and found both drugs to be equally effective in reducing delirium symptoms. In contrast, risperidone was associated with fewer extrapyramidal side effects [84], as reported in a Meta-analysis of 17 studies involving 1882 patients. Liu et al. demonstrated that haloperidol reduced the incidence of POD when used prophylactically in high-risk patients. Nevertheless, there was no significant improvement in duration or severity once delirium was established. Therefore, it is not recommended for routine prevention. However, it may be used cautiously for the treatment of acute agitation [85].

In the pathogenesis of postoperative delirium (POD), disturbances in cholinergic and dopaminergic neurotransmission play pivotal roles, with diminished acetylcholine activity and increased dopamine release contributing to attentional and cognitive disruption. Several pharmacologic trials have therefore explored strategies targeting these systems: for example, the use of acetylcholinesterase inhibitors (such as donepezil or rivastigmine) to boost cholinergic tone and dopamine-D_2_ receptor antagonists (such as haloperidol) to mitigate excessive dopaminergic signaling. One double-blind randomized controlled trial of intravenous physostigmine in elective liver surgery found no significant reduction in POD incidence (20% vs. 15%; *p* = 0.334) when administered post-induction for 24 h [86]. Systematic reviews similarly indicate that acetylcholinesterase inhibitors have not demonstrated consistent benefit in POD prevention (pooled RR ≈ 0.95; 95% CI 0.63–1.44) [87]. Nevertheless, the dopaminergic hypothesis is supported by clinical practice with D_2_ antagonists in the treatment of delirium and suggests a promising avenue for future prophylactic trials [88]. Future research should therefore examine combinations of cholinergic enhancement and dopaminergic modulation, potentially stratified by patient risk profiles and neurotransmitter biomarker status, to determine whether targeted pharmacologic interventions can reduce the incidence of POD by restoring neurotransmitter balance.

### Role of Dexmedetomidine

Dexmedetomidine is a highly selective α_2_-adrenoceptor agonist used to prevent and treat POD [89]. By binding α_2_-receptors, it suppresses norepinephrine release from the locus coeruleus via G protein-coupled pathways, reducing adenylyl cyclase activity and cAMP levels. It also exerts analgesia by blocking pain signals in A- and C-fibers, hyperpolarizing the descending noradrenergic spinal pathway, and inhibiting substance P release [90]. In POD pathophysiology, neuroinflammation drives microglial activation and proinflammatory cytokine release; dexmedetomidine attenuates this via α_2_-mediated suppression of IL-1 and TNF production, thereby preserving neuronal integrity [91,92]. It further inhibits inflammation in ischemia/reperfusion injury by blocking NF-κB and the TLR4/NF-κB pathway [93].

Surgical stress can trigger excessive glutamate release, intracellular Ca^2+^ overload, and ROS overproduction, leading to oxidative neuronal damage. Dexmedetomidine counters this by decreasing glutamate release and increasing BDNF levels, which support mitochondrial stability and reduce oxidative stress–induced neuronal apoptosis [94,95]. Its sedative, analgesic, anti-inflammatory, antioxidative, and neuroprotective effects are thoroughly documented. A review conducted by Bargnes et al. reported a 26% reduction in POD incidence with perioperative dexmedetomidine vs. 13% without it [80]. In a randomized controlled trial of 700 patients aged ≥ 65, low-dose postoperative dexmedetomidine reduced POD incidence from 23% to 9% [96]. A systematic review by Fondeur et al. confirmed its efficacy in ICU and elderly surgical populations, especially when given postoperatively [97]. However, Zorrilla-Vaca et al. found that intraoperative dexmedetomidine alone did not significantly reduce POD compared with placebo [98]. Overall, numerous trials and meta-analyses confirm that postoperative low-dose infusions of dexmedetomidine significantly reduce POD risk in the elderly, highlighting its anti-inflammatory and neuroprotective roles, particularly in ICU settings [99,100,101].

Dexmedetomidine has shown greater effectiveness in preventing postoperative delirium (POD) when administered during the postoperative period rather than intraoperatively. Intraoperative administration alone provides only a limited preventive benefit. This may be because many injurious processes—such as neuroinflammation, mitochondrial dysfunction, microglial activation, and blood–brain barrier (BBB) disruption—begin early during surgery and persist for hours to days after the operation [102]. Following the discussion of dexmedetomidine’s mechanistic effects, it is also essential to consider its plasma pharmacokinetics. The therapeutic plasma concentration of dexmedetomidine is approximately 0.2–3.2 ng/mL. Concentrations above 3 ng/mL are more likely to cause bradycardia, hypotension, and excessive sedation. Given its relatively short distribution half-life (~6 min) and elimination half-life (~2 h), plasma levels decline rapidly once the infusion stops. Consequently, the therapeutic effect on POD prevention diminishes quickly after discontinuation. Continuous or postoperative administration helps maintain effective concentrations during the critical period when neuroinflammatory and neurodegenerative processes are most active [103].

Future research on dexmedetomidine for the prevention of postoperative delirium (POD) should focus on optimizing timing, dosing, and mechanistic understanding. Extending infusion into the early postoperative period may better cover the window of neuroinflammation and BBB disruption. Studies should correlate plasma concentrations with POD outcomes to define adequate therapeutic levels and explore alternative administration routes for practicality. Incorporating biomarkers, neuroimaging, and EEG correlates could clarify how dexmedetomidine’s anti-inflammatory and neuroprotective actions translate into clinical benefit, guiding more targeted POD prevention strategies [103,104].

In summary, the effectiveness of dexmedetomidine stems from its multiple applications in the complex pathogenesis of POD. Thus, dexmedetomidine displays promising therapeutic potential for the efficient prevention or treatment of hyperactive-type POD. Future studies on pathogenesis and treatment methods must focus on complex investigations into the link between BBB injury and POCD development, as well as the search for new neuroprotective strategies. The review was written in a narrative format to cover a large amount of information and pay attention not only to prevention but also to various pathophysiological mechanisms, risk factors, assessment tools, etc. The limitation of the review lies in its narrative format, which addresses many facets but lacks the strengths of meta-analysis and systematic reviews. Those methods increase statistical power by pooling data across studies and reveal shared patterns that individual studies may not.

## 8. Conclusions

This review provides a comprehensive survey of the current knowledge on POD in elderly patients. The number of elderly patients undergoing surgery and anesthesia is expected to continue to increase. Along with the surgery, pre-existing comorbidities put them at a particular risk of developing POD and further POCD. POD remains a significant and under-recognized complication among elderly patients, being associated with increased morbidity, prolonged hospital stays, functional decline, and elevated mortality. POD has a complex, multifactorial pathogenesis that involves neuroinflammation, neurodegeneration, neuroimmune response, oxidative stress, drug-induced effects, vascular involvement, and potential glutamatergic involvement. Here, we demonstrated that type of anesthesia, depth of sedation, and hemodynamic stability can influence POD risk. Management should be multidisciplinary, with a primary emphasis on prevention through structured non-pharmacologic interventions, medication review, and optimizing intraoperative parameters. Pharmacologic treatment—when needed—should be tailored, with antipsychotics and dexmedetomidine used judiciously based on symptom severity and the patient profile. Thus, future research is required to explore how non-pharmacological and pharmacological interventions can be combined to reduce the risk of POD and the severity of cognitive decline. As the global population ages, reducing the burden of POD must become a central goal in perioperative geriatric care. Future research should focus on identifying reliable biomarkers, developing new pharmacological medications, and validating tailored intraoperative protocols to reduce the incidence of POD.

## Figures and Tables

**Table 1:** This table outlines the drug classes most commonly associated with a high risk of POD.

Drug Class	Drugs Examples	Mechanism	Delirium Risk
**Anticholinergics**	Diphenhydramine, Atropine, Scopolamine, Oxybutynin	Block central muscarinic receptors	**High**—strong link
**Benzodiazepines**	Lorazepam, Diazepam, Midazolam	Potentiate GABA-A → CNS depression	**High**—especially hypoactive POD
**Sedative-hypnotics**	Zolpidem, Eszopiclone	GABAergic sedation	*Moderate*
**Opioids**	Meperidine, Morphine, Hydromorphone	Dopaminergic and CNS depression	**High**—especially Meperidine
**Corticosteroids**	Prednisone, Dexamethasone	Neurostimulatory and mood-altering effects	*Moderate*
**H2-receptor antagonists**	Ranitidine, Famotidine	Cross BBB → confusion (especially in the renally impaired)	*Moderate*
**Antipsychotics (typical/atypical)**	Haloperidol, Risperidone, Olanzapine	Dopamine blockade	*Variable*—can cause or treat
**Tricyclic antidepressants**	Amitriptyline, Nortriptyline	Strong anticholinergic properties	**High**
**Antiepileptics**	Phenytoin, Carbamazepine	Altered neurotransmission	*Moderate*
**Muscle relaxants**	Cyclobenzaprine, Methocarbamol	Anticholinergic, sedative	**High**

## Data Availability

No new data were created or analyzed in this study. Data sharing is not applicable to this article.
